# Whole genome sequencing shows sleeping sickness relapse is due to parasite regrowth and not reinfection

**DOI:** 10.1111/eva.12338

**Published:** 2016-01-09

**Authors:** Joshua B. Richardson, Benjamin Evans, Patient P. Pyana, Nick Van Reet, Mark Sistrom, Philippe Büscher, Serap Aksoy, Adalgisa Caccone

**Affiliations:** ^1^ Department of Ecology and Evolutionary BiologyYale UniversityNew HavenCTUSA; ^2^ Department de ParasitologieInstitut National de Recherche BiomedicaleKinshasa GombeDemocratic Republic of the Congo; ^3^ Department of Biomedical SciencesInstitute of Tropical MedicineAntwerpBelgium; ^4^ School of Natural SciencesUniversity of California MercedMercedCAUSA; ^5^ Department of Epidemiology and Public HealthYale School of Public HealthNew HavenCTUSA

**Keywords:** drug resistance, human African trypanosomiasis, melarsoprol, population genomics, *Trypanosoma brucei gambiense*, whole genome sequencing

## Abstract

The trypanosome *Trypanosoma brucei gambiense* (*Tbg*) is a cause of human African trypanosomiasis (HAT) endemic to many parts of sub‐Saharan Africa. The disease is almost invariably fatal if untreated and there is no vaccine, which makes monitoring and managing drug resistance highly relevant. A recent study of HAT cases from the Democratic Republic of the Congo reported a high incidence of relapses in patients treated with melarsoprol. Of the 19 *Tbg* strains isolated from patients enrolled in this study, four pairs were obtained from the same patient before treatment and after relapse. We used whole genome sequencing to investigate whether these patients were infected with a new strain, or if the original strain had regrown to pathogenic levels. Clustering analysis of 5938 single nucleotide polymorphisms supports the hypothesis of regrowth of the original strain, as we found that strains isolated before and after treatment from the same patient were more similar to each other than to other isolates. We also identified 23 novel genes that could affect melarsoprol sensitivity, representing a promising new set of targets for future functional studies. This work exemplifies the utility of using evolutionary approaches to provide novel insights and tools for disease control.

## Introduction

Human African trypanosomiasis (HAT), also known as sleeping sickness, is endemic in many parts of sub‐Saharan Africa. The chronic and most prevalent form of the disease is caused by the protist *Trypanosoma brucei gambiense* (*Tbg*) in central and west Africa. There is no mammalian vaccine, and the disease is almost always fatal, if untreated. Few drugs are available to treat HAT, particularly once the trypanosomes have crossed the blood–brain barrier in the late stage of disease, and each comes with significant drawbacks. Melarsoprol is an arsenic‐based drug that, in the absence of any alternative, has been used to treat late stages of the disease for decades, despite causing encephalopathy in 6% of individuals, which is fatal 40% of the time (Blum et al. 2001). Today, the recommended first line therapy for late‐stage *gambiense* HAT is a combination of nifurtimox and eflornithine (Priotto et al. 2009; Simarro et al. 2012). Although these drugs are less toxic than melarsoprol, this combination therapy (NECT) is difficult to administer (Simarro et al. 2012), and resistance might soon be an issue, as up to 2% of cases relapse after NECT treatment (Franco et al. 2012).

Given this, and the absence of new drugs, melarsoprol is likely to remain in use for the foreseeable future. However, similar to many other drugs, melarsoprol resistance has become problematic. Beginning in the 1990s, several foci in HAT endemic regions have reported a high rate of melarsoprol treatment failure (Matovu et al. 2001a; Moore and Richer 2001; Stanghellini and Josenando 2001; Maina et al. 2007; Mumba Ngoyi et al. 2010). While melarsoprol treatment failure rate is typically less than 10%, areas in the Democratic Republic of the Congo (DRC) have reported rates as high as 58.8% (Moore and Richer 2001; Mumba Ngoyi et al. 2010). It is therefore critical to understand the reason for these failures. While several factors may contribute to the treatment failure rate, trypanosome resistance to melarsoprol is likely to be key.

Recently, a collection of 85 *Tbg* isolates from a hospital in the DRC reporting a high percentage of melarsoprol treatment failure was established (Mumba Ngoyi et al. 2010; Pyana et al. 2011). This collection includes strains isolated before and after treatment with melarsoprol from the same patients that had relapsed infections. These paired strains therefore present a unique opportunity to: (i) gain insights on the dynamics of relapses and (ii) systematically identify genetic determinants of melarsoprol resistance in natural *Tbg* strains. We sequenced the whole genomes of 19 *Tbg* isolates from this collection, including 4 pairs of strains derived from relapsed patients before and after melarsoprol treatment (Table 1). This enabled us to determine whether the strains isolated after treatment failure represent regrowth of the initial infecting strain, or alternatively, if they represent infections by a new strain. Taking advantage of this replicated design and a comparative approach, we analyze the patterns of genetic diversity among pre‐ and post‐treatment strains to make inferences on the evolutionary dynamics of the system, discuss the role of genetic variation in genes previously reported to be associated with melarsoprol sensitivity (Maser et al. 1999; Shahi et al. 2002; Alsford et al. 2012; Baker et al. 2012), and identify novel candidate genes that could affect melarsoprol sensitivity, thereby providing direction for future functional studies.

**Table 1:** Details of *Tbg* isolates from Mbuji‐Mayi, Democratic Republic of the Congo, used in this study

Intl. code	Patient number	Treatment outcome	Time of relapse (month)	Sampling point	Sample date	Sample source	Passage time (species, days)	Adapted to mice	Prior relapse	Treatment before inclusion	Treatment at inclusion
MHOM/CD/IN RB/2008/56	15	Cure		BT‐RE	30/05/05	CSF	G36G14	Y	N		M10
MHOM/CD/ST I/2006/01	45	Cure		BT	11/06/05	CSF	S13	Y	Y	M3, MN	E14
MHOM/CD/IN RB/2007/28	57	Relapse	12	AT	18/07/06	CSF	G49	Y	N		M10
MHOM/CD/IN RB/2008/62	85	Cure		BT	23/06/05	CSF	G7	Y	Y	M3	E14
MHOM/CD/IN RB/2008/64	141	Cure		BT	27/07/05	CSF	G9	Y	Y	M10	MN
MHOM/CD/IN RB/2005/02A	146	Relapse	3	BT	28/07/05	CSF	Mas38	Y	N		M10
MHOM/CD/IN RB/2006/05	146	Relapse	3	AT	10/11/05	Blood	Mas 4	Y	N		M10
MHOM/CD/IN RB/2005/01B	148	Relapse	3	BT	28/07/05	CSF	G5	Y	N		M10
MHOM/CD/IN RB/2006/14	148	Relapse	3	AT	5/01/06	CSF	Mas 16	Y	N		M10
MHOM/CD/IN RB/2006/06A	163	Relapse	3	AT	22/11/05	Blood	Mas 5	Y	Y	M3	MN
MHOM/CD/IN RB/2006/06A	163	Relapse	3	AT‐RE	22/11/05	Blood	Mas 5	Y	Y	M3	MN
MHOM/CD/IN RB/2006/21B	340	Relapse	3	AT	1/03/06	CSF	G11G7	Y	N		M10
MHOM/CD/IN RB/2006/22A	346	Relapse	3	BT	21/11/05	Blood	G30	N	N		M10
MHOM/CD/IN RB/2006/24B	346	Relapse	3	AT	3/03/06	CSF	G25G8	Y	N		M10
MHOM/CD/IN RB/2006/24B	346	Relapse	3	AT‐RE	3/03/06	CSF	G25G8	Y	N		M10
MHOM/CD/IN RB/2006/23A	348	Cure		BT	24/11/05	CSF	G13	Y	N		M10
MHOM/CD/IN RB/2006/16	349	Relapse	3	BT	25/11/05	CSF	G8	Y	N		M10
MHOM/CD/IN RB/2006/19	349	Relapse	3	AT	8/03/06	Blood	G7G5	Y	N		M10
MHOM/CD/IN RB/2008/34	378	Cure		BT	14/01/06	CSF	G21G9	Y	N		M10

International code: code of the stabilate. Patient number: number of the patient in the Mumba Ngoyi et al. (2010) study. Treatment outcome: treatment outcome of patient in the Mumba Ngoyi et al. (2010) study. Time of relapse (month): period after treatment when relapse was observed. Sampling point: BT, before treatment; AT, after treatment at moment of relapse. BT‐RE/AT‐RE, relapse in mouse carrying BT or AT sample and treated with melarsoprol. Sample date: date when blood or CSF specimen was taken and frozen in liquid nitrogen. Specimens stayed in liquid nitrogen until the first attempt to isolate the strain in *Grammomys surdaster* or *Mastomys natalensis* or severe combined immunodeficient (SCID) *Mus musculus)*. Sample source: blood or cerebrospinal fluid (CSF). Passage time (species, days): first letter of host (G. *surdaster, M. natalensis,* or SCID *M. musculus)* followed by days of infection before subpassage or before cryostabilization. Adapted to mice: whether or not the strain was adapted to laboratory mice after the isolation in *G. surdaster* or *M. natalensis*. Prior relapse: whether the patient was included in the Mumba Ngoyi et al. (2010) study as a patient that already experienced a relapse. Treatment before inclusion: M3: classic 3‐course treatment with melarsoprol, M10: abridged 10‐day treatment with melarsoprol, MN: melarsoprol–nifurtimox combination therapy, Treatment at inclusion: as above plus P8: 8 days pentamidine, E14: 14 days eflornithine.

## Materials and methods

### Samples

Information about the Tbg strains used in this study is summarized in Table 1. All the clinical *Tbg* samples came from the cryobank of the World Health Collaboration Center for Research and Training on Human African Trypanosomiasis Diagnostics and originated from patients enrolled in a longitudinal study of HAT at Dipumba Hospital in Mbuji‐Mayi, DRC. Details of this study are in Mumba Ngoyi et al. (2010) and the methods of trypanosome isolation and adaptation to mouse hosts are described in detail in Pyana et al. (2011). To summarize the methods found in these studies, blood or cerebrospinal fluid (CSF) containing trypanosomes was taken from the patient when they were first admitted, before treatment (BT). Patients were then given three types of treatment: a 10‐day melarsoprol regimen (M10), a combination of melarsoprol and nifurtimox, or a 14‐day eflornithine regimen (E14). If the patient subsequently relapsed and was readmitted, another sample was taken (AT). Trypanosomes were preserved by immediately freezing blood or CSF mixed with a cryomedium in liquid nitrogen. To establish a stable, rodent‐adapted trypanosome line, samples were thawed and inoculated in one of three rodent species: *Grammomys surdaster*, immuno‐suppressed *Mastomys natalensis*, or immuno‐deficient *Mus musculus*. Trypanosome strains were maintained in hosts, by subpassaging if necessary, until parasite density reached 10^6.9^/mL . At this point, a blood sample was drawn and frozen in liquid nitrogen. This cryostabilized sample was then thawed and injected into a *M. musculus* host. Once parasite density reached 10^7.8^/mL, the trypanosome isolate was considered adapted to the mouse host. All but one isolate used in this study (346 BT) were adapted to mice. Mouse‐adapted strains were given four doses of melarsoprol (10 mg/kg). If the mouse relapsed after this treatment, a blood sample was drawn and trypanosomes isolated. These samples were labeled AT‐RE or BT‐RE, depending on if the original isolate came from a patient before (BT) or after (AT) treatment. DNA from all isolates was extracted using a Qiagen DNA blood kit (Venlo, Netherlands).

### Sequence and clustering analysis

DNA extracted from trypanosome isolates was submitted to the Yale Center for Genome Analysis for sequencing on the Illumina Hi‐Seq 2000 platform (San Diego, CA, USA). Quality of the reads was checked through FastQC (Andrews 2010). In addition to the 19 *Tbg* clinical samples, we included in this study raw sequencing data from the *Tbg* strain 1829‐Aljo (Sistrom et al. 2014), which was used for comparison, as it was isolated in the 1970s from a patient in the DRC.

Raw reads were aligned to the large chromosomes of the *Tbg* DAL 972 reference genome (Jackson et al. 2010), using Bowtie2 (Langmead and Salzberg 2012). Single nucleotide polymorphisms (SNPs) were called using the samtools (v1.1) mpileup, using the default quality cutoff (Li et al. 2009). Only SNPs with a minimum read depth of 10 were retained. SNP calling was also performed using the Genome Analysis Toolkit (GATK) (McKenna et al. 2010). Realignment around indels and base quality recalibration was performed following the best practices guidelines (Van der Auwera et al. 2013). For SNPs obtained from both pipelines, SNPs with minor allele frequency less than 0.05 were discarded. RepeatMasker (Smit et al. 2013–2015) was used to identify repetitive elements in the *Tbg* DAL972 reference genome, and any SNPs found in these elements were eliminated. Coding sequences of variant surface glycoprotein (VSG) genes in the DAL972 genome were identified using gene text search function of the tritrypdb.org website (Aslett et al. 2010). SNPs appearing in these sequences were eliminated, as the highly repetitive nature of VSG genes would make accurate SNP calling difficult. The ape (3.0‐11) package in R (v.3.0.3) was used to conduct a cluster analysis and construct a neighbor‐joining tree with 10 000 bootstrap replicates from the filtered set of SNPs (Jombart et al. 2010; Jombart and Ahmed 2011). Chi‐square tests comparing the frequency of heterozygous SNPs between patient pairs were performed using the chisq.test command in R. *F*_ws_ was calculated following Manske et al. (2012). The filtered set of SNPs was placed in 10 bins, according to their MAF. The heterozygosity at each SNP (equal to 1 − ($F_{1}^{2}+F_{2}^{2}$), where *F*_1_ and *F*_2_ are the allele frequencies) for the population as a whole and for each individual sample was calculated. For each bin, the average heterozygosity of the sample was plotted against the average heterozygosity of the population at each bin, and a linear regression was performed in R. The *F*_ws_ for each sample was calculated as 1 minus the slope of the regression.

To complement the clustering analyses, we also carried out a multivariate analysis, discriminant analysis of principal components (DAPC) using the R package adegenet (1.3–9.2) (Jombart et al. 2010). This method constructs principal components (synthetic combinations of variables, in this case SNP genotypes) that best differentiate two or more groups of individuals. As a first step in the DAPC analysis, we used *k*‐means clustering with the SNP data set transformed by principal component analysis for a range of values of *k* (the putative number of clusters). Examination of these results indicated 4 to be a reasonable value of *k*. To avoid over‐fitting of data, we retained two principal components following optimization procedures as recommended by the package developers (Jombart et al. 2010).

### Candidate gene identification

Genes previously implicated in melarsoprol resistance were identified in Alsford et al. (2012). Each of the eight genes listed as a ‘primary’ hit in Supplementary data file 1 for melarsoprol resistance was used as a query to search for homologs in the *Tbg* genome, using the BLAST tool at the tritrypdb.org website (Aslett et al. 2010). We also investigated four genes, *TbAT1*,* TbAQP2, TbAQP3,* and *TbMRPA*, which have been shown previously to affect melarsoprol sensitivity (Maser et al. 1999; Shahi et al. 2002; Baker et al. 2012).

To identify novel candidate melarsoprol resistance genes, the adegenet package in R was used for discriminant analysis of principal components (DAPC) on the set of SNPs used in the clustering analysis above. We performed the DAPC analyses on two data sets. One analysis grouped the strains using their isolation status (BT or AT, Table 1) and included the four pairs of strains isolated from the same patient as a prior grouping. The other analysis was conducted only on the 9 BT strains placing them in cured (four strains) and noncured (five strains) groups. For each data set (patient pairs and cured versus noncured), DAPC was performed on 100 randomized versions of the data set to estimate the frequency of SNPs expected at various loading values by chance. In these simulations, SNP genotypes at each loci were randomly sampled without replacement. This analysis generated a distribution of loading values of SNPs that differentiated two groups of strains with arbitrary genotypes and allowed us to estimate the frequency of SNPs expected at various loading values by random chance. A false discovery rate was estimated by comparing the number of SNPs at a given loading value identified by the real and randomized DAPC analyses. SNPs with a loading value equal to or above the loading value that minimized the FDR were used to identify genes that could be involved in melarsoprol resistance. Increasing the number of randomized data sets to 120 did not change the estimate of the minimized FDR.

The program SNPeff (Cingolani et al. 2012) was used to identify any *Tbg* genes with candidate SNPs occurring in their coding sequence, and the corresponding protein changes caused by it (nsSNPs), if any. We searched for homologs of these genes in *Tbb* through the BLAST function at TriTrypDB.org (Aslett et al. 2010). We used data from Jensen et al. (Jensen et al. 2009), accessed through the TriTrypDB.org (Aslett et al. 2010) website, to check whether a *Tbb* homolog of the candidate gene is expressed in the bloodstream form of *Tbb*. The same homologous gene names were searched for in the *Tbg* bloodstream‐form expression data from Veitch et al. (2010), as these data were mapped to the *Tbb* genome (Veitch et al. 2010). The web interfaces of PolyPhen‐2 (Adzhubei et al. 2010) and SNAP (Bromberg and Rost 2007) were used to predict effects of changes on protein function. We report PolyPhen2 results based on the algorithm trained on the HumDiv data set, which is considered to be more accurate than that based on the algorithm trained on the HumVar data set (Adzhubei et al. 2010). We used MESSA, the meta server for sequence analysis to identify functional domains in genes of unknown function (Cong and Grishin 2012).

## Results

### Sequencing of strains isolated from HAT patients in DRC

**Figure 1:**
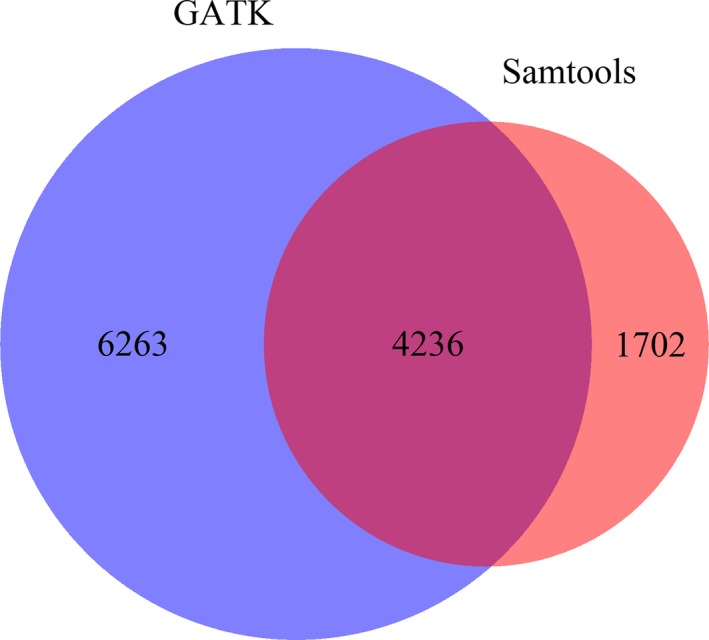
Overlap between single nucleotide polymorphisms (SNPs) identified by Genome Analysis Toolkit (GATK) Haplotype Caller and Samtools mpileup. Number of genomic positions containing SNPs according to samtools mpileup (pink), GATK (purple), or both (red).

We recovered an average of 18.2 million reads from each *Tbg* isolate, with an average of 80.5% of reads mapping to the *Tbg* reference strain, DAL972 (Jackson et al. 2010), and an average coverage of 40.5 ± 7.6 (SD). To enable genomewide comparisons of these strains, we identified SNPs with a minimum read depth of 10, using both the samtools mpileup command and the GATK Haplotype Caller tool (Li et al. 2009; McKenna et al. 2010). The samtools mpileup command called 22 201 SNPs, and the Haplotype Caller identified 47 054 SNPs. For both sets, we filtered out SNPs with a minor allele frequency less than 0.05, located in a repetitive region of the genome, or in the coding sequences of VSG genes. This left a final set of 5938 SNPs called by Samtools and 10 499 SNPs called by Haplotype Caller. These SNP sets overlap at 4236 positions (Fig. 1). As the samtools set appears to be more conservative, and 71% of the SNPs in this set were also called by the GATK Haplotype Caller, we report the analysis from the samtools set, unless otherwise noted.

### Strains isolated from the same patient are most similar

**Figure 2:**
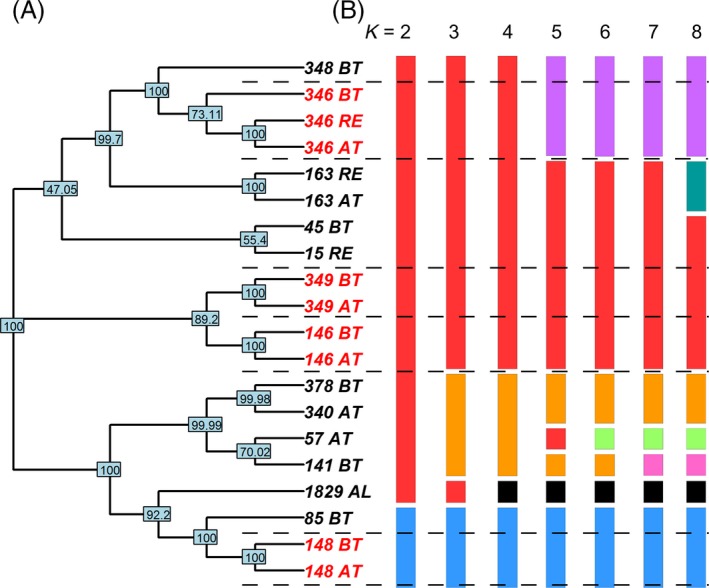
Cluster analysis of *Tbg* isolates. (A) Neighbor‐joining tree of *Tbg* isolates based on 5938 single nucleotide polymorphisms (SNPs) and 10 000 replicates obtained using the ape package in R. Bootstrap percentages are shown on the nodes. Red strains come from patients where both a BT and an AT strain are part of the data set. 346AT‐RE isolated from mouse carrying 346AT which relapsed after being treated with melarsoprol. 1829 Aljo is a *Tbg* strain isolated from the DRC in the 1970s, included for comparison. (B) Strains grouped by *k*‐means clustering of SNP principal components (Jombart et al. 2010). For *k* values from 2 through 8, group membership for each strain is shown by color.

Figure 2A shows a neighbor‐joining network based on clustering of the 5938 SNPs found in the 19 *Tbg* patient derived strains (Table 1) and, for comparison, 1829 Aljo, a *Tbg* strain isolated in DRC in the 1970s. The pairs of strains isolated from the same patients before (BT) and after treatment (AT) are sister taxa to each other, implying that they are more closely related to each other than to any of the other strains. These pairs do not cluster together, but instead are scattered throughout the tree. High bootstrap support at these nodes strengthens this result. The only relatively minor and expected exception is the relationship of the three 346 strains. From this patient, a sample was taken after treatment (346 AT) and subsequently tested for melarsoprol resistance in a mouse. Another isolate was taken from the mouse (346 AT‐RE) when found to be resistant. In the cluster analysis, 346 AT and 346 AT‐RE are sister taxa, with the 346 BT isolate being the first one to link to the clade with these two strains.

**Figure 3:**
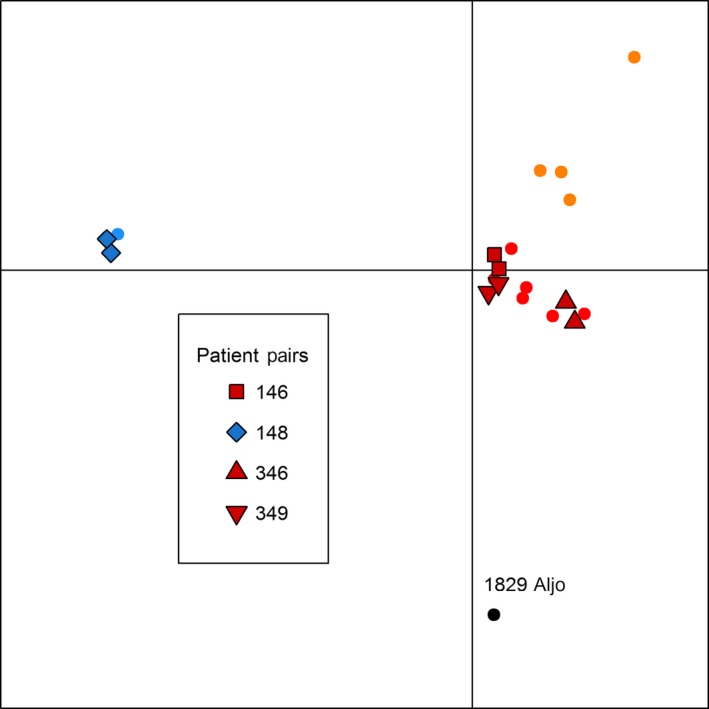
Discriminant analysis of principal components (DAPC) Scatter plot of *Tbg* isolates. First two discriminant functions from DAPC analysis of *Tbg* isolates obtained using the DAPC function from the adegenet package in R. Group membership is shown by color while strains isolated from the same patient share the same shape. Circles represent the 14 other *Tbg* isolates included in this study and 1829 Aljo. The shape and color of the four patient‐pair strains are detailed in the insert.

In addition to the neighbor‐joining analysis, we performed a DAPC on the same set of 5938 SNPs. Typically, the *k* value with the lowest Bayesian information criterion (BIC) is thought to most accurately reflect the number of groups present in the sample. In our case, *k* values from 1 through 6 have similar BIC values (Figure S1). Group assignments from *k*‐means clustering of SNP genotype principal components (Fig. 2B) are similar to the patterns observed in the neighbor‐joining network (Fig. 2A). Similar to the clustering analysis shown in Fig. 1, each paired strain from the same patient belongs to the same cluster in the first two discriminant functions of the DAPC at *k* = 4 (Fig. 3). As expected, the 1829 Aljo strain is distinct from the others. This same general pattern holds for *k* values from 2 to 8 (Fig. 2B). Both neighbor‐joining and DAPC methods indicate that strains derived from the same patient are genetically more similar to each other than to other strains. Qualitatively similar results were obtained using the set of SNPs obtained by GATK (Figure S2). The one exception is that strain 346 BT is sister to 348 BT, although the node containing these strains is sister to the node containing 346 AT.

### Strains isolated from the same patient do not differ in heterozygosity

As multiple trypanosome strains can co‐occur, with a multiple infection frequency as high as 47% in some areas (MacLeod et al. 1999; Balmer and Caccone 2008), exposure to melarsoprol could select for resistant strains among those infecting the patient. This could result in the apparent reduction of heterozygosity when comparing samples before and after treatment from the same patient, because a sample comprised of multiple genetic strains presumably has a higher frequency of apparent heterozygosity relative to a sample comprised of a single strain. Table S1 reports the results of chi‐square tests for a difference in heterozygosity between the BT and AT sample for each patient pair. There was no statistically significant difference in the proportion of heterozygous SNPs between the BT and AT pairs. In addition, we computed *F*_ws_, a measure of a sample's heterozygosity relative to the population as a whole, while controlling for minor allele frequency (Manske et al. 2012). The *F*_ws_ is largely similar between strains isolated before and after treatment (Figure S3). This suggests AT strains are just as diverse as their BT counterparts. If the level of heterozygosity is due to the presence of multiple strains, the same mix of strains must have been maintained throughout treatment.

### Variation in genes previously shown to affect melarsoprol resistance

Table 2 summarizes variation found in 14 genes known to be associated with melarsoprol resistance from previous studies (Maser et al. 1999; Shahi et al. 2002; Alsford et al. 2012; Baker et al. 2012). As reported in Pyana Pati et al. (2014), these isolates contain a chimeric gene, *TbAQP2/3*, in place of the *TbAQP2* and *TbAQP3* loci, and are wild type at the *TbAT1* locus. Our results support this finding. Also, strain 1829 Aljo was found to be wild‐type at *TbAQP2, TbAQP3*, and the *TbAT1* loci. Three genes, *TbMRPA*,* Tbg972.10.1740,* and *upstream binding protein 1* (*UBP1*), have nonsynonymous SNPs (nsSNPs). All of the patient‐paired strains are heterozygous for the nsSNPs in *UBP1* and *Tbg972.4.240*. Only one gene (*Tbg972.10.1740*) shows a heterozygous difference between a patient pair (148). The function of this gene is unknown, but the mutation is rated as ‘possibly damaging’ by the mutation effect prediction program PolyPhen2 (Adzhubei et al. 2010). Apart from this gene, there is very little variation among the strains in the previously known candidate melarsoprol sensitivity genes.

**Table 2:** Previously identified candidate melarsoprol genes

Gene number	Protein name/des cription	nsSNPs	Amino acid change	Predicted effect		Strain Genotypes							
						146		148		346		349	
				PolyPhen2	SNAP	BT	AT	BT	AT	BT	AT	BT	AT
Tbg972.2.2440	Trypanothione synthetase				No nsSNPs								
Tbg972.4.2470	Multidrug resistance‐associated protein (MRPA)	C → G	T469R	Benign	Neutral	C/G	C/G	C/G	C/G	C/G	C/G	C/G	C/G
–	–	A → G	N752D	Benign	Neutral	A/G	A/G	A/G	A/G	A/G	A/G	A/G	A/G
Tbg972.5.40	Adenosine transporter 1 (AT1)				No nsSNPs								
Tbg972.6.1170	Aquaporin 1				No nsSNPs								
Tbg972.8.5950	Protein kinase				No nsSNPs								
Tbg972.9.80	Hypothetical protein				No nsSNPs								
Tbg972 10 1740	Hypothetical protein	G → T	C379F	Benign	Neutral	G/T	G/T	G/T	G/T	G/T	G/T	G/T	G/T
–	–	G → A	D507F	Possibly damaging	Neutral	G/G	G/G	G/G	G/A	G/G	G/G	G/G	G/G
Tbg972.10.2310	Putative serine/threonine protein kinase				No nsSNPs								
Tbg972.10.14510	Mapk11 homolog				No nsSNPs								
Tbg972.10.16540	Aquapglycero‐porin 2 (TbAQP2 homolog)				Chimeric allele with Tbg972.10.16560								
Tbg972.10.16560	Aquapglycero‐porin 2 (TbAQP3 homolog)				Chimeric allele with Tbg972.10.16540								
Tbg972.10.18650	Hypothetical protein				No nsSNPs								
Tbg972.10.19640	Mapk11 homolog				No nsSNPs								
Tbg972.11.450	Upstream binding protein 1 (UBP1)	T → G	S204A	Unknown	Neutral	T/G	T/G	T/G	T/G	T/G	T/G	T/G	T/G

Genetic variation found in the 14 genes previously known to affect melarsoprol sensitivity. Any nsSNPs observed are listed, along with the protein change caused by the nsSNP and its predicted effect on protein function. PolyPhen2: result based on the HumDiv data set, SNAP: results from the SNAP web interface. Genotypes of patient‐pair strains are listed in the final eight columns. Italicized genotypes indicate a heterozygous difference between strains isolated from the same patient. – Indicates same entry as line above.

### Identification of novel candidate genes that could influence melarsoprol resistance

**Figure 4:**
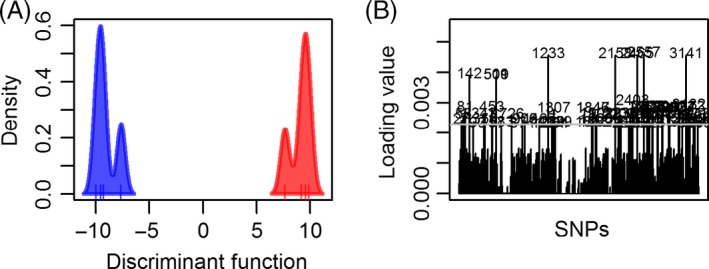
Significant single nucleotide polymorphisms (SNPs) Identified by DAPC (A) Discriminant function values separating BT (Red) and AT (Blue) strains obtained using the Discriminant analysis of principal component function in the adegenet package of R. The *Y* axis represents discriminant function values, and the *X* axis represents density of individual strains. (B) Loading values of individual SNPs contributing to the discriminant function shown in A. SNPs are shown on the *Y* axis, and loading values on the *X* axis. Values above line are significantly higher than expected by chance.

We used two methods of identifying candidate genes that affect melarsoprol sensitivity. First, we used DAPC to identify SNPs that distinguish BT and AT strains in the WGS data. We restricted this analysis to the four BT/AT pairs, as we were interested in looking for parallel variation that occurred in multiple pairs. We performed the DAPC dividing these strains into two groups (four AT and four BT strains) using the set of 5,938 SNPs called by samtools, described above. The discriminant function completely distinguishes the two groups (Fig. 4A), and Fig. 4B shows the loading values of each SNP contributing to this discriminant function. To evaluate these results, we calculated a false discovery rate (FDR) by performing DAPC analyses on data sets with randomly assigned genotypes to estimate the distribution of loading values contributing to the discriminant function by chance. This enabled us to calculate the FDR by comparing the number of SNPs at a given loading value in real versus simulated data. We identified 69 SNPs with a loading value over 0.00229 (Fig. 4B), minimizing the false discovery rate at 13% (Fig. S4). We chose this cutoff value to ensure we report the largest proportion of SNPs likely to be true positives. While this loading value is low, this may simply reflect that many genes contribute melarsoprol resistance, and/or that other nongenetic factors also play a significant role.

In addition to these 69 SNPs, we also identified 53 SNPs with a homozygous, fixed difference between at least one of the patient pairs (e.g. A/A in a BT strain changed to G/G in the AT strain), as these differences are likely to have more severe phenotypic consequences than heterozygous ones. Six of these 53 SNPs were also identified in the DAPC screen. In total, we identified 122 unique SNPs with genotypes differentiating between BT and AT strains (Table S2).

Out of these 122 differentiating SNPs, we found 35 nsSNPs in 23 genes (Table 3). Although none of these genes was previously known to be involved in melarsoprol sensitivity (Table 2), one of them, *Tbg972.10.18680*, is highly similar to, and approximately 4 kb upstream of, a previously known candidate gene, *Tbg972.10.18650*. Sixteen of the 23 genes are expressed in the bloodstream form of *Tbg* (Veitch et al. 2010; Table 3). Expression could not be determined for three of them, because they do not have an obvious homolog in *Trypanosoma brucei brucei* (*Tbb*), which was used as the reference genome in Veitch et al. (2010) (listed as ‘unknown’ in Table 3). To gain insights in to the potential functional relevance of SNPs in these genes, we used two software programs that provide predictions of the effects of the mutation on the encoded protein, Polyphen2 (Adzhubei et al. 2010) and SNAP (Bromberg and Rost 2007). The results are shown in Table 3, columns 8 and 9. Seven genes, *expression site‐associated gene 2* (*ESAG2*), *Tbg972.3.4800*,* Tbg972.9.9130*,* Tbg972.8.7620*,* Tbg972.10.1360*,* Tbg972.10.19330*, and *Tbg972.11.4390*, contain a SNP with a ‘nonbenign’ or non‐neutral effect on the encoded protein.

**Table 3:** Novel candidate genes

Gene number	Protein name/description	nsSNP	Protein change	Predicted effect		Blood‐stream expression	Strain genotypes							
							146		148		346		349	
				PolyPhen2	SNAP		BT	AT	BT	AT	BT	AT	BT	AT
Tbg972.1.2860	Hypothetical protein	A → G	N270D	Unknown	Neutral	Yes	*G/G*	*A/G*	*G/G*	*A/G*	***G/G***	A/A	A/G	A/G
Tbg972.3.4800	Hypothetical protein	T → C	L33P	Probably damaging	Non‐neutral	Unknown	*T/T*	*T/C*	*T/T*	*T/C*	***T/T***	***C/C***	T/T	T/T
–	–	C → T	L34F	Benign	Non‐neutral	–	*C/C*	*C/T*	*C/C*	*C/T*	***C/C***	***T/T***	C/C	C/C
–	–	C → G	Q35V	Benign	Non‐neutral	–	*C/C*	*C/G*	*C/C*	*C/G*	***C/C***	***G/G***	C/C	C/C
–	–	A → T	Q35V	–	–	–	*A/A*	*A/T*	A/A	A/A	***A/A***	***T/T***	A/A	A/A
–	–	C → G	L40V	Benign	Neutral	–	C/C	C/C	C/C	C/C	***C/C***	***G/G***	C/C	C/C
–	–	G → A	C43Y	Benign	Non‐neutral	–	*G/A*	*G/G*	G/G	G/G	***G/G***	***A/A***	*G/A*	G/G
Tbg972.4.5180	Hypothetical protein	A → G	V199A	Benign	Neutral	Yes	A/G	A/G	***A/A***	***G/G***	*A/A*	A/G	*A/G*	*A/A*
Tbg972.5.370	Hypothetical invariant surface glycoprotein	G → A	G141N	Benign	Neutral	No	***G/G***	***A/A***	A/A	A/A	A/A	A/A	A/A	A/A
–	–	G → A	G141N	–	–	–	***G/G***	***A/A***	*G/A*	*A/A*	A/A	A/A	A/A	A/A
Tbg972.5.6250	Hypothetical protein	G → A	V16I	Failed	Neutral	Unknown	G/A	G/A	*G/A*	*G/G*	*G/A*	*G/G*	*G/A*	*G/G*
Tbg972.6.700	Metacaspase 3 (MCA3)	C → T	V124I	Benign	Neutral	Yes	C/T	C/T	***T/T***	***C/C***	*C/C*	*C/T*	*C/T*	*C/C*
Tbg972.7.7504	Hypothetical protein	G → A	L116F	Failed	Neutral	Unknown	*G/A*	*G/G*	*G/A*	*G/G*	*G/A*	*G/G*	G/G	G/G
Tbg972.7.8020	Putative ex‐pression site associated gene (ESAG)	G → C	E49Q	Benign	Neutral	Yes	C/C	C/C	***G/G***	***C/C***	C/C	C/C	***G/G***	***C/C***
Tbg972.8.7620	Hypothetical protein	T → C	F491S	Probably damaging	Non‐neutral	Yes	T/T	T/T	T/T	T/T	***T/T***	***C/C***	T/T	T/T
Tbg972.8.7910	Amino acid transporter 1	C → T	T219I	Benign	Neutral	No	C/C	C/C	*C/T*	*C/C*	*C/T*	*C/C*	*C/T*	*C/C*
Tbg972.9.9130	Putative leucine‐rich repeat protein (LRRP)	C → T	P220L	Benign	Neutral	No	C/C	C/C	C/C	C/C	***T/T***	***C/C***	C/C	C/C
–	–	C → T	A358V	Benign	Neutral	–	C/C	C/C	***T/T***	***C/C***	***C/C***	***T/T***	C/C	C/C
–	–	T → G	F364C	Benign	Neutral	–	G/G	G/G	***G/G***	***T/T***	***T/T***	***G/G***	G/G	G/G
–	–	G → C	G1335R	Probably damaging	Non‐neutral	–	*G/C*	*G/G*	*G/C*	*G/G*	*G/C*	*G/G*	G/C	G/C
Tbg972.10.1150	Hypothetical protein	T → C	S22G	Benign	Neutral	Yes	*T/C*	*T/T*	*T/C*	*T/T*	*T/C*	*T/T*	*T/C*	*T/T*
Tbg972.10.1360	Putative serine carboxypeptidase III precur sor	C → A	F409L	Possibly damaging	Non‐neutral	No	C/C	C/C	C/C	C/C	C/C	C/C	***A/A***	***C/C***
Tbg972.10.18680	Hypothetical protein	T → G	F92C	Failed	Neutral	Yes	T/T	T/T	*T/G*	*G/G*	*T/G*	*G/G*	*T/G*	*G/G*
Tbg972.10.19330	Hypothetical protein	C → G	A644G	Benign	Non‐neutral	Yes	*C/G*	*C/C*	*C/C*	*C/G*	***G/G***	***C/C***	*C/G*	*G/G*
Tbg972.10.19940	Putative expression site associated gene (ESAG)	T → A	K395I	Benign	Neutral	Yes	***T/T***	***A/A***	***A/A***	***T/T***	A/A	A/A	A/A	A/A
Tbg972.11.1610	Putative leucine‐rich repeat protein (LRRP)	T → G	C435G	Benign	Neutral	Yes	***G/G***	***T/T***	G/G	G/G	G/G	G/G	G/G	G/G
Tbg972.11.4390	Hypothetical protein	C → T	R17W	Probably damaging	Non‐neutral	Yes	C/C	C/C	T/T	T/T	***T/T***	***C/C***	***T/T***	***C/C***
Tbg972.11.4390	Hypothetical protein	A → G	Y38C	Benign	Non‐neutral	‐‐	A/A	A/A	A/A	A/A	***A/A***	***G/G***	A/A	A/A
Tbg972.11.10190	Hypothetical protein	G → A	G766S	Unknown	Neutral	Yes	G/G	*G/A*	G/A	G/A	*G/G*	*G/A*	*G/G*	*G/A*
Tbg972.11.11950	Hypothetical protein	C → T	P2365S	Unknown	Neutral	Yes	C/T	C/T	C/T	*C/T*	*C/T*	*T/T*	***C/C***	***T/T***
Tbg972.11.12410	Hypothetical protein	G → A	A321T	Benign	Neutral	Yes	G/G	G/G	*G/G*	*G/A*	*G/G*	*G/A*	*G/G*	*G/A*
Tbg972.11.16370	Expression site‐associated gene 2 (ESAG2)	G → A	D351S	Possibly damaging	Neutral	Yes	G/A	G/A	*G/A*	*G/G*	*G/A*	*G/G*	*G/A*	*G/G*
–	–	A → G	D351S	–	–	–	A/G	A/G	*A/G*	*A/A*	*A/G*	*A/A*	*A/G*	*A/A*
–	–	G → C	G352A	Benign	Neutral	–	G/C	G/C	*G/C*	*G/G*	*G/C*	*G/G*	*G/C*	*G/G*
Tbg972.11.19250	Expression site‐associated gene 4 (ESAG4)	T → A	S390T	Benign	Neutral	Yes	T/A	T/A	*T/A*	*T/T*	*T/A*	*T/T*	*T/A*	*T/T*

Genetic variation found in 23 genes containing nsSNPs identified by our screen. Gene Number: gene number in DAL972 genome. Protein name/description: name of protein or brief description, if known. Observed nsSNPs are listed, along with the protein change caused by the nsSNP and its predicted effect on protein function. PolyPhen2: result based on the HumDiv data set, SNAP: results from the SNAP web interface. Expressed in Tbg bloodstream form: data from Veitch et al. (2010). Genotypes of patient‐pair strains are listed in the final eight columns. Italicized genotypes indicate a heterozygous difference between strains isolated from the same patient. Bold italicized genotypes indicate a fixed, homozygous difference between strains isolated from the same patient. – Indicates same entry as line above.

To identify genetic variants present prior to treatment that could have affected melarsoprol sensitivity and thus allow some BT strains to resist treatment, we carried out a DAPC analysis on all nine strains classified as BT in Table 1. We divided the strains into two groups: one consisting of 5 strains isolated from patients that were ultimately cured, and the other consisting of the 4 BT strains from the patient pairs, which were not cured. Although the discriminant function did not distinguish the cured and relapsed BT strains well (Figure S5), we found 76 SNPs with a loading value greater than 0.0015 corresponding to a minimized FDR of 0.337 (Figure S6). These SNPs are summarized in Table S4.

## Discussion

Reconstructing the historical path of adaptation and the genes involved in it is a difficult exercise. Selection can affect patterns of variation in the genome in different ways, and it is difficult to distinguish from background variation, random drift, and gene flow. Further, evidence continues to mount for the importance of standing variation to adaptation in natural populations, but detecting selection on standing variation is even harder than on *de novo* mutations (Messer and Petrov 2013). In such cases, one way to facilitate the distinction between neutral vs. adaptive changes is to take advantage of natural replicates and to look at the parallel distribution of changes among them. Neutral changes brought about by gene drift or gene flow should be affecting all gene regions similarly, while adaptive ones should result in parallel patterns in different replicates. This evolutionary approach has been very effective in identifying selective patterns and candidate for association studies in a variety of organisms (Schoville et al. 2012).

We took advantage of this comparative approach combined with whole genome sequencing to study the evolutionary history underlying the occurrence of melarsoprol treatment failure among *Tbg* strains, comparing variation in replicate pairs to identify candidate gene regions that could be associated with this trait. By looking at the clustering patterns of the BT/AT patient pairs in relation to each other and to other strains in the region, we could reconstruct the evolutionary history of the emergence of melarsoprol resistance and evaluate if trypanosome isolates from the same patient obtained before and after melarsoprol treatment represent the same strain, and not multiple independently acquired infections. Multiple clustering methods clearly support this hypothesis, as isolates from the same patient are more similar to each other than to other isolates (Fig. 2). This suggests that melarsoprol resistance has been acquired independently by the different AT strains. If this is correct, this finding has two important epidemiological implications: (i) relapsing patients were infected with a strain initially sensitive to melarsoprol that subsequently accumulated the functional mutations necessary for resistance in the patient and (ii) the evolution of resistance for melarsoprol could occur through multiple paths involving different genomic regions as the likelihood of two identical mutations occurring independently in four different replicates is quite small.

Admittedly, the plausibility of this scenario is difficult to assess without an accurate estimate of mutation rates in trypanosomes, which is not currently available. However, we argue this is the most likely possibility given the alternatives. If patients were reinfected after initial treatment, it seems unlikely they would coincidentally be reinfected by a strain most similar to the originally infecting strain. Another alternative is that the initial infection could have included multiple strains, providing the standing variation necessary to select for melarsoprol resistance. We also argue that this is unlikely because, although multiple infections are relatively common in other vertebrate hosts and in the tsetse fly vector, they are much less common in humans (Balmer and Caccone 2008; Aksoy et al. 2013). This is possibly due to a severe bottleneck during the parasite transfer through the tsetse bite or interstrain competition in the human blood. Regardless of its likelihood, if melarsoprol resistance was due to standing variation and infection by multiple strains, we would expect a decrease in heterozygosity, reflecting a loss of variation as one strain out‐competes the rest. This is not the pattern we observe, as strains isolated BT have similar levels of heterozygosity as the strains isolated after treatment (Table S1), making this scenario unlikely.

By looking at the clustering pattern of the BT strains, our results also shed light on the diversity of strains that can survive melarsoprol treatment as we have five strains from the same geographic area from patients that were ultimately cured after treatment and others that relapsed. The clustering analysis shows that the BT strains isolated from patients that were ultimately cured (Table 1) do not tend to cluster together (Fig. 2). This suggests that the emergence of resistance can occur from different genetic backgrounds, as there is not a single lineage that is resistant or sensitive. Instead, strains from multiple genetic backgrounds in this region have the potential to survive treatment, as opposed to a single resistant strain explaining drug treatment failures. This has important epidemiological implications and can also explain why we find multiple genetic variants in different genes associated with melarsoprol resistance in the replicate BT/AT pairs (see below).

Whole genome sequencing and the replicate pair design also enabled us to investigate patterns of variation in loci known to be involved in melarsoprol resistance and to search for additional candidate loci. A number of genes have already been identified as candidate melarsoprol resistance genes, which impact several pathways. Laboratory strains of *Tbg* resistant to melarsoprol have been found to be defective in the P2 adenosine transport system (Carter and Fairlamb 1993). The gene encoding the adenosine transporter was identified as *TbAT1* and mutations in this gene resulted in reduced uptake of melarsoprol (Maser et al. 1999). An RNAi screen identified additional candidate genes conferring resistance to antitrypanocidal drugs when knocked down, and established a role for aquaglyceroporins in both melarsoprol resistance and melarsoprol–pentamidine cross‐resistance (pentamidine is a drug commonly used to treat early stages of HAT).

Naturally, occurring mutations in *TbAT1* have been found in strains infecting patients in Uganda that relapsed after melarsoprol treatment (Matovu et al. 2001b). However, among the melarsoprol resistant strains, some had wild‐type *TbAT1* alleles, suggesting that other factors are also involved (Matovu et al. 2001b). Furthermore, strains isolated from a region in Sudan with high melarsoprol treatment failure rate did not appear to have resistant *TbAT1* alleles (Maina et al. 2007). Similar to *TbAT1*, mutations in aquaglyceroporins occur in natural populations, but are not sufficient to predict relapse after melarsoprol treatment (Graf et al. 2013; Pyana Pati et al. 2014). Many potential targets of mutation appear to exist that can alter a strain's sensitivity, increasing the likelihood an infection will persist through treatment. However, no single genotype appears to confer resistance to all strains. Consistent with this observation, each of the 19 *Tbg* isolates in our study, regardless of patient treatment outcome, contain a chimeric *TbAQP2/3* gene, which is associated with increased pentamidine and melarsoprol cross‐resistance (Baker et al. 2012; Pyana Pati et al. 2014).

Our results support the hypothesis that melarsoprol resistance is polygenic in nature (Alsford et al. 2012). In our own set of candidate genes, only the genotypes at *Tbg972.10.1150* completely distinguish BT and AT strains (Table 3, line 21). The BT strains are heterozygous at this position, while the AT strains are homozygous for the reference allele. It therefore seems unlikely that variation in this gene completely explains melarsoprol sensitivity in the strains examined. Further, the genotypes at no SNPs completely distinguish strains isolated from patients that were ultimately cured from those that relapsed. We were thus unable to find a consistent genotypic pattern that predicted melarsoprol sensitivity, even within the region of the DRC where our trypanosome samples were isolated.

The 23 genes identified by our comparative genomic approach (Table 3) represent novel genes that could play a role in melarsoprol resistance. Currently, very little phenotypic data are available for these genes. Even though expression data for *Tbg* is lacking, most of the candidate genes have some evidence of expression in the bloodstream form of a group 2 *Tbg* strain (Table 3, Veitch et al. 2010). While many candidate genes are lacking annotations, the fact that the genes are expressed, even in a strain outside the main *Tbg* lineage, suggests the genes have some functionality (and are not, e.g., pseudogenes). The Veitch et al. (2010) experiment mapped reads to only 81% of the genes in the *Tbb* genome, possibly explaining why some genes in Table 3 do not appear to be expressed (Veitch et al. 2010). Three of the candidates in Table 3 make especially compelling candidates for further study.

**Figure 5:**
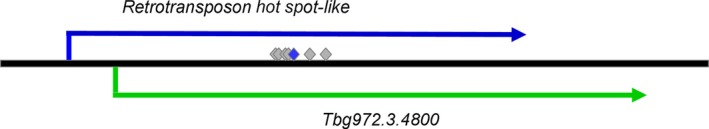
Single nucleotide polymorphisms (SNPs) in the Tbg972.3.4800 locus. Diagram showing the *Tbg972.3.4800* locus, including the *Tbg972.3.4800* open reading frame (orf), and another putative orf encoding a retrotransposon hot spot (RHS)‐like protein. Gray diamonds represent SNPs identified in our screen, and the blue diamond represents a SNP that eliminates a stop codon found in the DAL972 reference strain, allowing read through of the RHS‐like orf.

*ESAG2* is of interest because it contains three nsSNPs, resulting in a potentially disruptive change to the expressed protein. The *Tbg972.3.4800* locus contains a cluster of 7 SNPs identified by our screen as important in distinguishing BT from AT strains, and may encode a trypanosome‐specific retrotransposon hot spot protein (Bringaud et al. 2002; Fig. 5). *Tbg972.10.18680*, and the nearby locus *Tbg972.10.18650*, also warrant further examination. *Tbg972.10.18650* is homologous to the *Tbb* gene *Tb927.10.15310*, which was identified in an RNAi screen for candidate genes that affect melarsoprol sensitivity (Alsford et al. 2012). *Tbg972.10.18680* is approximately 4 kb upstream of *Tbg972.10.18650* and consists of an open reading frame 366 base pairs long, which is similar to part of the open reading frame of *Tbg972.10.18650*. Although the exact function of these genes is unknown, the identification of two highly similar loci as candidates affecting melarsoprol sensitivity suggests a more detailed investigation of these genes is warranted (See Appendix S1 for further discussion).

Because relapsing strains tend to cluster with the corresponding initially infecting strains, and few mutations are shared between the relapsed strains, it is likely that a combination of factors which may be parasite and/or patient specific determine the failure or success of melarsoprol treatment. Although our screen of candidate genes was fairly extensive for the strains at our disposal, it should be noted that it was not exhaustive, as we sampled a subset of the naturally circulating strains from one geographic region and only used strains that could be cultured in rodents (Pyana et al. 2011; Table 1). While whole genome sequencing of additional strains from relapsed patients may reveal novel loci influencing melarsoprol sensitivity in those strains, this study identified several novel candidate genes which should be targeted for further functional investigations to better understand the mechanistic basis of melarsoprol resistance emergence.

Although it is important to stress that many factors (e.g., patient's genotype and behavior, disease stage, variation in gene expression, interaction between host and parasite genomes) in addition to genetic variation in one or more parasite genes involved in determining resistance to melarsoprol, our results highlight the utility of using next‐gene sequencing and evolutionary and comparative approaches to address problems of clinical and epidemiological relevance. We show that trypanosome strains with different genetic backgrounds from a region of high incidence of melarsoprol failure regrow after initial treatment causing disease relapse, that many loci from known and novel genes involved in melarsoprol resistance contribute to an individual strain's sensitivity to melarsoprol, and point to future functional work on novel genes that could lead to innovative treatments.

## Data archiving statement

Data available from the Dryad Digital Repository: http://dx.doi.org/10.5061/dryad.d58b8b.

## Supporting information


**Appendix S1.** Candidate genes.

**Figure S1.** Cluster identification.
**Figure S2.** Cluster analysis of *Tbg* isolates.
**Figure S3.** SNP Heterozygosity.
**Figure S4.** False discovery rate of SNPs distinguishing patient Pairs.
**Figure S5.** Significant SNPs Identified by DAPC.

**Table S1.** Proportion of heterozygous SNPs in patient pairs.
**Table S2.** List of SNPs that differentiate BT and AT strains.
**Table S3.** List of SNPs that differentiate cured and relapsing strains.

## References

[eva12338-bib-0001] Adzhubei, I. A. , S. Schmidt , L. Peshkin , V. E. Ramensky , A. Gerasimova , P. Bork , A. S. Kondrashov et al. 2010 A method and server for predicting damaging missense mutations. Nature Methods 7:248–249. 20354512 10.1038/nmeth0410-248 PMC2855889

[eva12338-bib-0002] Aksoy, S. , A. Caccone , A. P. Galvani , and L. M. Okedi 2013 Glossina fuscipes populations provide insights for human African trypanosomiasis transmission in Uganda. Trends in Parasitology 29:394–406. 23845311 10.1016/j.pt.2013.06.005 PMC3772539

[eva12338-bib-0003] Alsford, S. , S. Eckert , N. Baker , L. Glover , A. Sanchez‐Flores , K. F. Leung , D. J. Turner et al. 2012 High‐throughput decoding of antitrypanosomal drug efficacy and resistance. Nature 482:232–236. 22278056 10.1038/nature10771 PMC3303116

[eva12338-bib-0500] Andrews, S. 2010 FastQC a quality control tool for high throughput sequence data. Available from http://www.bioinformatics.babraham.ac.uk/projects/fastqc/ (accessed on 24 September 2013).

[eva12338-bib-0004] Aslett, M. , C. Aurrecoechea , M. Berriman , J. Brestelli , B. P. Brunk , M. Carrington , D. P. Depledge et al. 2010 TriTrypDB: a functional genomic resource for the Trypanosomatidae. Nucleic Acids Research 38:D457–D462. 19843604 10.1093/nar/gkp851 PMC2808979

[eva12338-bib-0005] Baker, N. , L. Glover , J. C. Munday , D. Aguinaga Andres , M. P. Barrett , H. P. de Koning , and D. Horn 2012 Aquaglyceroporin 2 controls susceptibility to melarsoprol and pentamidine in African trypanosomes. Proceedings of the National Academy of Sciences of the United States of America 109:10996–11001. 22711816 10.1073/pnas.1202885109 PMC3390834

[eva12338-bib-0006] Balmer, O. , and A. Caccone 2008 Multiple‐strain infections of *Trypanosoma brucei* across Africa. Acta Tropica 107:275–279. 18671933 10.1016/j.actatropica.2008.06.006 PMC2582348

[eva12338-bib-0007] Blum, J. , S. Nkunku , and C. Burri 2001 Clinical description of encephalopathic syndromes and risk factors for their occurrence and outcome during melarsoprol treatment of human African trypanosomiasis. Tropical Medicine and International Health 6:390–400. 11348533 10.1046/j.1365-3156.2001.00710.x

[eva12338-bib-0008] Bringaud, F. , N. Biteau , S. E. Melville , S. Hez , N. M. El‐Sayed , V. Leech , M. Berriman et al. 2002 A new, expressed multigene family containing a hot spot for insertion of retroelements is associated with polymorphic subtelomeric regions of *Trypanosoma brucei* . Eukaryotic Cell 1:137–151. 12455980 10.1128/EC.1.1.137-151.2002 PMC118050

[eva12338-bib-0009] Bromberg, Y. , and B. Rost 2007 SNAP: predict effect of non‐synonymous polymorphisms on function. Nucleic Acids Research 35:3823–3835. 17526529 10.1093/nar/gkm238 PMC1920242

[eva12338-bib-0010] Carter, N. S. , and A. H. Fairlamb 1993 Arsenical‐resistant trypanosomes lack an unusual adenosine transporter. Nature 361:173–176. 8421523 10.1038/361173a0

[eva12338-bib-0011] Cingolani, P. , A. Platts , L. Wang le , M. Coon , T. Nguyen , L. Wang , S. J. Land et al. 2012 A program for annotating and predicting the effects of single nucleotide polymorphisms, SnpEff: SNPs in the genome of Drosophila melanogaster strain w1118; iso‐2; iso‐3. Fly (Austin) 6:80–92. 22728672 10.4161/fly.19695 PMC3679285

[eva12338-bib-0012] Cong, Q. , and N. V. Grishin 2012 MESSA: MEta‐server for protein sequence analysis. BMC Biology 10:82. 23031578 10.1186/1741-7007-10-82 PMC3519821

[eva12338-bib-0014] Franco, J. R. , P. P. Simarro , A. Diarra , J. Ruiz‐Postigo , M. Samo , and J. Jannin 2012 Monitoring the use of nifurtimox‐eflornithine combination therapy (NECT) in the treatment of second stage gambiense human African trypanosomiasis. Research and Reports in Tropical Medicine 3:93–101. 10.2147/RRTM.S34399 PMC6067772 30100776

[eva12338-bib-0015] Graf, F. E. , P. Ludin , T. Wenzler , M. Kaiser , R. Brun , P. P. Pyana , P. Buscher et al. 2013 Aquaporin 2 mutations in *Trypanosoma brucei gambiense* field isolates correlate with decreased susceptibility to pentamidine and melarsoprol. PLoS Neglected Tropical Diseases 7:e2475. 24130910 10.1371/journal.pntd.0002475 PMC3794916

[eva12338-bib-0016] Jackson, A. P. , M. Sanders , A. Berry , J. McQuillan , M. A. Aslett , M. A. Quail , B. Chukualim et al. 2010 The genome sequence of *Trypanosoma brucei gambiense*, causative agent of chronic human African trypanosomiasis. PLoS Neglected Tropical Diseases 4:e658. 20404998 10.1371/journal.pntd.0000658 PMC2854126

[eva12338-bib-0017] Jensen, B. C. , D. Sivam , C. T. Kifer , P. J. Myler , and M. Parsons 2009 Widespread variation in transcript abundance within and across developmental stages of *Trypanosoma brucei* . BMC Genomics 10:482. 19840382 10.1186/1471-2164-10-482 PMC2771046

[eva12338-bib-0018] Jombart, T. , and I. Ahmed 2011 adegenet 1.3‐1: new tools for the analysis of genome‐wide SNP data. Bioinformatics 27:3070–3071. 21926124 10.1093/bioinformatics/btr521 PMC3198581

[eva12338-bib-0019] Jombart, T. , S. Devillard , and F. Balloux 2010 Discriminant analysis of principal components: a new method for the analysis of genetically structured populations. BMC Genetics 11:94. 20950446 10.1186/1471-2156-11-94 PMC2973851

[eva12338-bib-0021] Langmead, B. , and S. L. Salzberg 2012 Fast gapped‐read alignment with Bowtie 2. Nature Methods 9:357–359. 22388286 10.1038/nmeth.1923 PMC3322381

[eva12338-bib-0022] Li, H. , B. Handsaker , A. Wysoker , T. Fennell , J. Ruan , N. Homer , G. Marth et al. 2009 The Sequence Alignment/Map format and SAMtools. Bioinformatics 25:2078–2079. 19505943 10.1093/bioinformatics/btp352 PMC2723002

[eva12338-bib-0024] MacLeod, A. , C. M. Turner , and A. Tait 1999 A high level of mixed *Trypanosoma brucei* infections in tsetse flies detected by three hypervariable minisatellites. Molecular and Biochemical Parasitology 102:237–248. 10498180 10.1016/s0166-6851(99)00101-2

[eva12338-bib-0025] Maina, N. , K. J. Maina , P. Maser , and R. Brun 2007 Genotypic and phenotypic characterization of *Trypanosoma brucei* gambiense isolates from Ibba, South Sudan, an area of high melarsoprol treatment failure rate. Acta Tropica 104:84–90. 17765860 10.1016/j.actatropica.2007.07.007

[eva12338-bib-0026] Manske, M. , O. Miotto , S. Campino , S. Auburn , J. Almagro‐Garcia , G. Maslen , J. O'Brien et al. 2012 Analysis of *Plasmodium falciparum* diversity in natural infections by deep sequencing. Nature 487:375–379. 22722859 10.1038/nature11174 PMC3738909

[eva12338-bib-0027] Maser, P. , C. Sutterlin , A. Kralli , and R. Kaminsky 1999 A nucleoside transporter from *Trypanosoma brucei* involved in drug resistance. Science 285:242–244. 10398598 10.1126/science.285.5425.242

[eva12338-bib-0028] Matovu, E. , J. C. Enyaru , D. Legros , C. Schmid , T. Seebeck , and R. Kaminsky 2001a Melarsoprol refractory T. b. gambiense from Omugo, north‐western Uganda. Tropical Medicine and International Health 6:407–411. 11348535 10.1046/j.1365-3156.2001.00712.x

[eva12338-bib-0029] Matovu, E. , F. Geiser , V. Schneider , P. Maser , J. C. Enyaru , R. Kaminsky , S. Gallati et al. 2001b Genetic variants of the TbAT1 adenosine transporter from African trypanosomes in relapse infections following melarsoprol therapy. Molecular and Biochemical Parasitology 117:73–81. 11551633 10.1016/s0166-6851(01)00332-2

[eva12338-bib-0030] McKenna, A. , M. Hanna , E. Banks , A. Sivachenko , K. Cibulskis , A. Kernytsky , K. Garimella et al. 2010 The Genome Analysis Toolkit: a MapReduce framework for analyzing next‐generation DNA sequencing data. Genome Research 20:1297–1303. 20644199 10.1101/gr.107524.110 PMC2928508

[eva12338-bib-0031] Messer, P. W. , and D. A. Petrov 2013 Population genomics of rapid adaptation by soft selective sweeps. Trends in Ecology and Evolution 28:659–669. 24075201 10.1016/j.tree.2013.08.003 PMC3834262

[eva12338-bib-0032] Moore, A. , and M. Richer 2001 Re‐emergence of epidemic sleeping sickness in southern Sudan. Tropical Medicine and International Health 6:342–347. 11348529 10.1046/j.1365-3156.2001.00714.x

[eva12338-bib-0033] Mumba Ngoyi, D. , V. Lejon , P. Pyana , M. Boelaert , M. Ilunga , J. Menten , J. P. Mulunda et al. 2010 How to shorten patient follow‐up after treatment for *Trypanosoma brucei gambiense* sleeping sickness. Journal of Infectious Diseases 201:453–463. 20047500 10.1086/649917

[eva12338-bib-0034] Priotto, G. , S. Kasparian , W. Mutombo , D. Ngouama , S. Ghorashian , U. Arnold , S. Ghabri et al. 2009 Nifurtimox‐eflornithine combination therapy for second‐stage African *Trypanosoma brucei gambiense* trypanosomiasis: a multicentre, randomised, phase III, non‐inferiority trial. Lancet 374:56–64. 19559476 10.1016/S0140-6736(09)61117-X

[eva12338-bib-0035] Pyana Pati, P. , N. Van Reet , D. Mumba Ngoyi , I. Ngay Lukusa , S. Karhemere Bin Shamamba , and P. Buscher 2014 Melarsoprol sensitivity profile of *Trypanosoma brucei gambiense* isolates from cured and relapsed sleeping sickness patients from the Democratic Republic of the Congo. PLoS Neglected Tropical Diseases 8:e3212. 25275572 10.1371/journal.pntd.0003212 PMC4183442

[eva12338-bib-0036] Pyana, P. P. , I. Ngay Lukusa , D. Mumba Ngoyi , N. Van Reet , M. Kaiser , S. Karhemere Bin Shamamba , and P. Buscher 2011 Isolation of *Trypanosoma brucei gambiense* from cured and relapsed sleeping sickness patients and adaptation to laboratory mice. PLoS Neglected Tropical Diseases 5:e1025. 21526217 10.1371/journal.pntd.0001025 PMC3079580

[eva12338-bib-0039] Schoville, S. D. , A. Bonin , O. François , S. Lobreaux , C. Melodelima , and S. Manel 2012 Adaptive genetic variation on the landscape: methods and cases. Annual Review of Ecology, Evolution, and Systematics 43:23–43.

[eva12338-bib-0040] Shahi, S. K. , R. L. Krauth‐Siegel , and C. E. Clayton 2002 Overexpression of the putative thiol conjugate transporter TbMRPA causes melarsoprol resistance in *Trypanosoma brucei* . Molecular Microbiology 43:1129–1138. 11918801 10.1046/j.1365-2958.2002.02831.x

[eva12338-bib-0041] Simarro, P. P. , J. Franco , A. Diarra , J. A. Postigo , and J. Jannin 2012 Update on field use of the available drugs for the chemotherapy of human African trypanosomiasis. Parasitology 139:842–846. 22309684 10.1017/S0031182012000169

[eva12338-bib-0501] Sistrom, M. , B. Evans , R. Bjornson , W. Gibson , O. Balmer , P. Maser , S. Aksoy , et al. 2014 Comparative genomics reveals multiple genetic backgrounds of human pathogenicity in the Trypanosoma brucei complex. Genome Biology and Evolution 6:2811–2819. 25287146 10.1093/gbe/evu222 PMC4224348

[eva12338-bib-0502] Smit, A. F. A. , R. Hubley , and P. Green 2013–2015. RepeatMasker Open‐4.0. Available from http://www.repeatmasker.org (accessed on 11 February 2014).

[eva12338-bib-0042] Stanghellini, A. , and T. Josenando 2001 The situation of sleeping sickness in Angola: a calamity. Tropical Medicine and International Health 6:330–334. 11348527 10.1046/j.1365-3156.2001.00724.x

[eva12338-bib-0043] Van der Auwera, G. A. , M. O. Carneiro , C. Hartl , R. Poplin , G. Del Angel , A. Levy‐Moonshine , T. Jordan et al. 2013 From FastQ data to high confidence variant calls: the Genome Analysis Toolkit best practices pipeline. Current Protocols in Bioinformatics 11:1–33. 25431634 10.1002/0471250953.bi1110s43 PMC4243306

[eva12338-bib-0044] Veitch, N. J. , P. C. Johnson , U. Trivedi , S. Terry , D. Wildridge , and A. MacLeod 2010 Digital gene expression analysis of two life cycle stages of the human‐infective parasite, *Trypanosoma brucei gambiense* reveals differentially expressed clusters of co‐regulated genes. BMC Genomics 11:124. 20175885 10.1186/1471-2164-11-124 PMC2837033

