# Supplementary material for: Whole genome sequencing shows sleeping sickness relapse is due to parasite regrowth and not reinfection
Source: Evol Appl. 2016 Jan 9;9(2):381–93. doi: 10.1111/eva.12338 (PMC4721075; doi:10.1111/eva.12338)
Supplement: Supplementary file 1 — Appendix S1. Candidate genes. [file EVA-9-381-s001.docx]

**Appendix S1. Candidate Genes**

*ESAG2* contains a variant surface glycoprotein domain, found in other ESAG and VSG genes (Reddy et al. 1990) and three nsSNPs that occur in two adjacent codons (Table 3, lines 32-34). Collectively, these changes were classified as “possibly damaging” by PolyPhen2. The BT strains from patients 148, 346, and 349 are heterozygous for the mutant genotype, while the AT strains from these patients are homozygous (both 146 BT and AT are heterozygous for the mutant genotype).

*Tbg972.3.4800* contains a cluster of six fixed nsSNPs between the 346AT/BT pair, (Table 3, lines 2-7). This gene has no orthologs in other trypanosome species listed at TriTrypDB, and its function is unknown. However, there is an open reading frame starting 28 bp upstream of the *Tbg972.3.4800* orf which encodes a protein similar to retrotransposon hot spot (RHS) proteins found in other trypanosomes (Fig. 5). Interestingly, in the reference strain (DAL972) this orf contains a stop codon after 34 amino acids, in nine of the strains in this study this stop codon is mutated to glutamic acid, allowing the full-length transcript to be expressed.

Gene *Tbg972.9.9130* encodes a putative protein containing a leucine-rich repeat domain, which are typically involved in protein-protein interactions (Kobe and Deisenhofer 1994), and contains a nsSNP (Table 3, line 20) likely to have a non-neutral effect according to SNAP. The BT strains in patient 146, 148, and 346 are heterozygous at this location, while the AT strains are homozygous for the neutral wild-type allele. Both 349AT and 349BT are heterozygous at this position.

References:

Reddy, L. V., T. Hall, and J. E. Donelson 1990. Sequences of three VSG mRNAs expressed in a mixed population of *Trypanosoma brucei* rhodesiense. Biochemical and biophysical research communications **169**:730–736.

Kobe, B., and J. Deisenhofer 1994. The leucine-rich repeat: a versatile binding motif. Trends in Biochemical Science **19**:415–421.
