# Supplementary figures and images for: Whole genome sequencing shows sleeping sickness relapse is due to parasite regrowth and not reinfection
Source: Evol Appl. 2016 Jan 9;9(2):381–93. doi: 10.1111/eva.12338 (PMC4721075; doi:10.1111/eva.12338)

Supplemental Figures

Figure S1

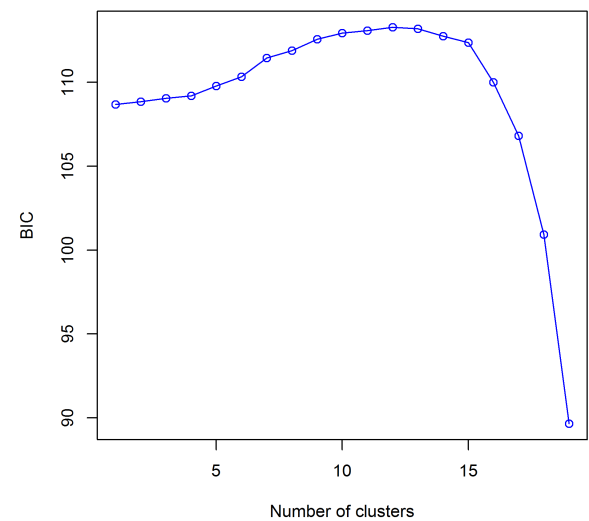

Figure S2

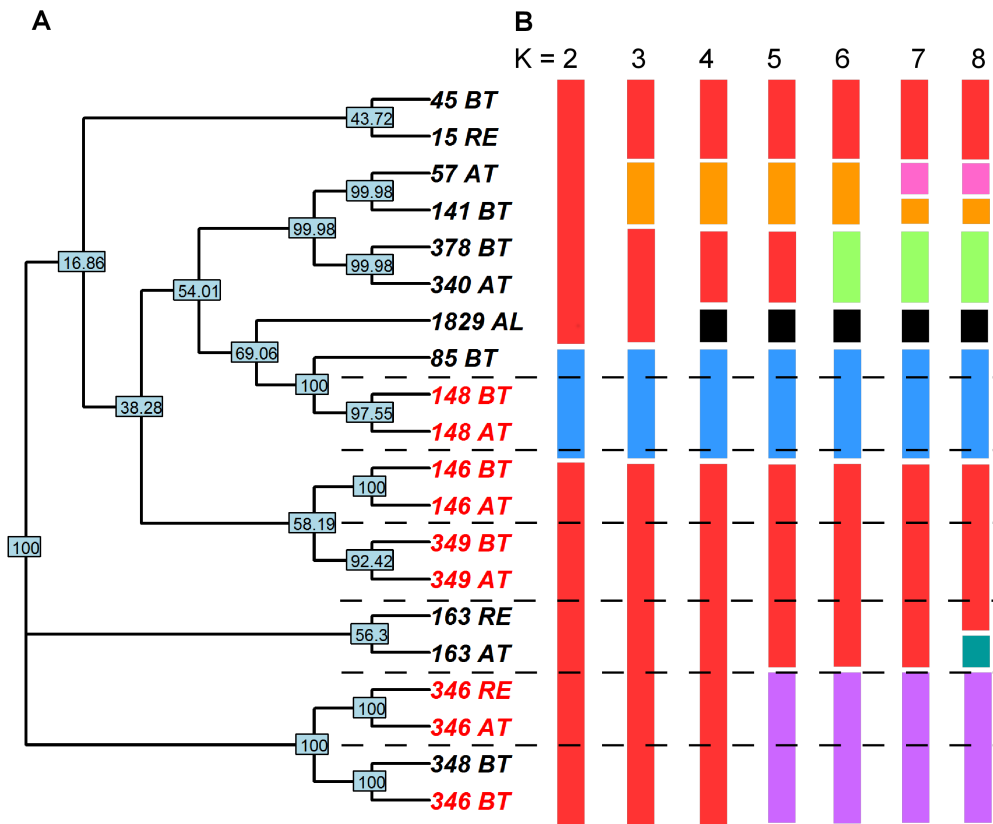

Figure S3

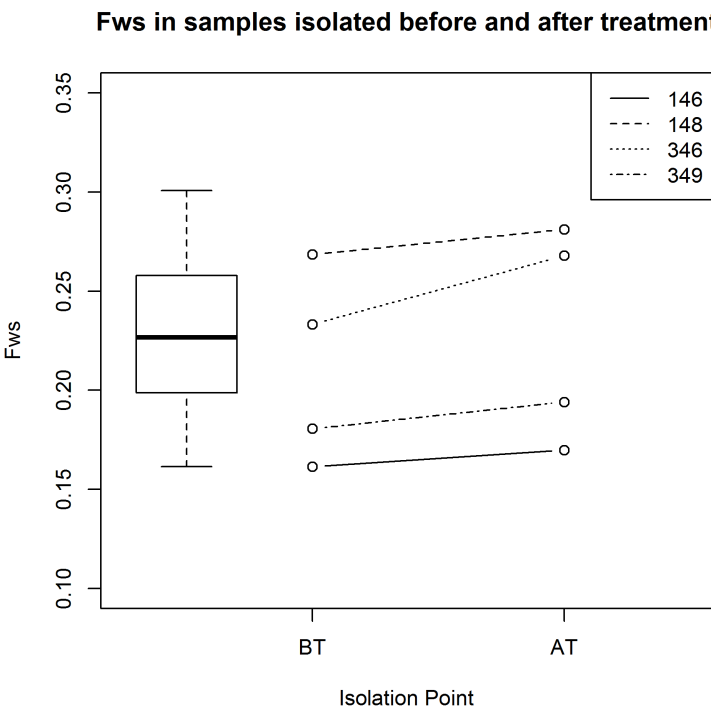

Figure S4

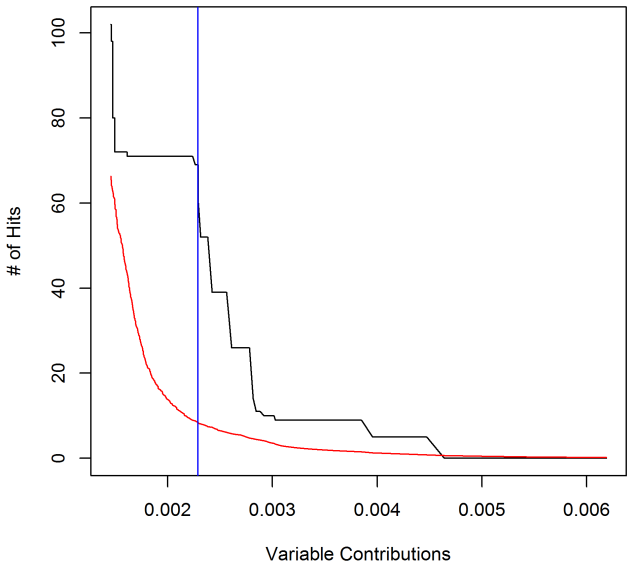

### Figure S6

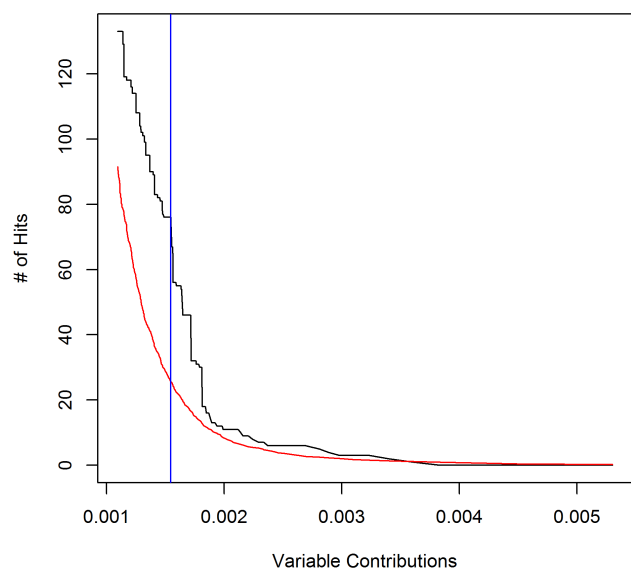

Supplement: Supplementary file 2 — Figure S1. Cluster identification. Figure S2. Cluster analysis of Tbg isolates. Figure S3. SNP Heterozygosity. Figure S4. False discovery rate of SNPs distinguishing patient Pairs. Figure S5. Significant SNPs Identified by DAPC. [file EVA-9-381-s002.pdf]
