# Supplementary material for: Whole genome sequencing shows sleeping sickness relapse is due to parasite regrowth and not reinfection
Source: Evol Appl. 2016 Jan 9;9(2):381–93. doi: 10.1111/eva.12338 (PMC4721075; doi:10.1111/eva.12338)
Supplement: Supplementary file 3 — Table S1. Proportion of heterozygous SNPs in patient pairs. Table S2. List of SNPs that differentiate BT and AT strains. Table S3. List of SNPs that differentiate cured and relapsing strains. [file EVA-9-381-s003.docx]

**Supplementary Tables**

**Table S1: Proportion of heterozygous SNPs in patient pairs.** Number of heterozygous and homozygous SNPs in each strain belonging to a patient pair. **a** P values from a chi-square test for a difference in proportion of heterozygous SNPs between strains isolated from the same patient.

**Table S2:** **List of SNPs that differentiate BT and AT strains.** Loading Rank: Rank based on loading value of SNP contributing to discriminant function, SNP Position: Coordinates in DAL972 genome (Jackson et al. 2010), WT: SNP genotype in DAL972 genome, MUT: Alternative SNP genotype, Patient Pair with a Homozygous Difference: Patient pair with a homozygous difference in SNP genotype, Coding SNP: If the SNP occurs in a coding sequence, nsSNP: If the SNP encodes a non-synonymous change, Gene: If a coding SNP, the coordinates of the gene in the DAL972 genome.

**Table S3:**  **List of SNPs that differentiate cured and relapsing strains.** Loading Rank: Rank based on loading value of SNP contributing to discriminant function, SNP Position: Coordinates in DAL972 genome (Jackson et al. 2010), WT: SNP genotype in DAL972 genome, MUT: Alternative SNP genotype, Patient Pair with a Homozygous Difference: Patient pair with a homozygous difference in SNP genotype, Coding SNP: If the SNP occurs in a coding sequence, nsSNP: If the SNP encodes a non-synonymous change, Gene: If a coding SNP, the coordinates of the gene in the DAL972 genome.
